# Beyond the dental chair: determinants of parental acceptance of behavior management techniques in pediatric dentistry

**DOI:** 10.3389/froh.2026.1837896

**Published:** 2026-08-20

**Authors:** Apitchaya Pruksametanan, Kornkan Phuengnam, Abedelmalek Kalefh Tabnjh, Siddharthan Selvaraj

**Affiliations:** 1 Institute of Dentistry, Suranaree University of Technology, Nakhon Ratchasima, Thailand; 2 Institute of Nursing, Suranaree University of Technology, Nakhon Ratchasima, Thailand; 3 The Integrated Transdisciplinary Research Unit for Sustainable Well-Being, Suranaree University of Technology, Nakhon Ratchasima, Thailand; 4 Department of Cardiology, Institute of Odontology, The Sahlgrenska Academy, University of Gothenburg, Gothenburg, Sweden; 5 Department of Applied Dental Sciences, Faculty of Applied Medical Sciences, Jordan University of Science and Technology, Irbid, Jordan; 6 Department of Pathology, Saveetha Medical College and Hospital, Saveetha Institute of Medical and Technical Sciences, Chennai, India; 7 Faculty of Dentistry, University of Puthisastra, Phnom Penh, Cambodia; 8 Department of Dental Research Cell, Dr. D. Y. Patil Dental College & Hospital, Dr. D. Y. Patil Vidyapeeth (Deemed to be University), Pune, India

**Keywords:** behavior management techniques, dental anxiety, pediatric dentistry, parental acceptance, parenting styles

## Abstract

**Background:**

Developing positive dental experiences at a young age will go a long way toward building a foundation for positive oral health in the future. Pediatric dentists routinely employ behavior management techniques (BMTs) to help children cope with the process of receiving dental care; however, parental acceptance of these methods plays an important role in determining their effectiveness. There are many sociodemographic and psychosocial characteristics that could influence a parent's view on specific management strategies. This study explored some of the variables associated with parental acceptance of both basic and advanced BMTs among Thai parents in the Nakhon Ratchasima province.

**Methods:**

A cross-sectional study was conducted from June to October 2025 with parents or primary caregivers of children receiving dental treatment at the Oral Health Center of Suranaree University of Technology and selected preschools located within the Muang district of Thailand. Participants provided their opinions using a structured questionnaire. Questions measuring sociodemographic characteristics, parental dental anxiety (Modified Dental Anxiety Scale), family role approach, and acceptance or rejection of nine types of BMTs were included in the questionnaire. The scaled response items were measured using a five-point Likert scale. Descriptive statistics for each measure were compiled from independent-samples *t*-tests, Welch analysis of variance, and *post hoc* tests, using significance levels of *p* < 0.05.

**Results:**

In total, 215 parents participated in the study. Female parents exhibited significantly higher levels of acceptance of participatory communication methods with their child (i.e., Ask–Tell–Ask, Parent' Presence/Absence) (*p* < 0.05). In addition, higher levels of education, increased anxiety about dental treatment, and an authoritative style of parenting were associated with increased levels of acceptance of communicative techniques. Younger parents were more accepting of the Tell–Show­–Do technique of communication than older parents.

**Conclusion:**

Parental acceptance of behavioral management techniques was influenced by both demographic and psychosocial variables. Once pediatric dentists understand these associated factors, they can tailor their methods of communication, thereby improving the overall experience of the child receiving dental care.

## 1 Introduction

A child's lifelong oral health can be influenced by past dental experiences. Establishing positive dental experiences in childhood lays the foundation for lifelong oral health. In pediatric dentistry, many behavior management techniques (BMTs) have been developed and taught, with the primary goal transcends mere clinical success. Successful outcomes in pediatric dentistry involve not only the delivery of best practice but also the fostering of a child's ability to cope with dental environment with a positive attitude. However, the dental environment is often considered aversive by young patients. Complex dental procedures involving injections and prolonged procedure time are a source of intense anxiety, which can compromise the quality of dental care and lead to poor prognosis (1). Without appropriate behavior management, these early stressful dental exposures may result in a lifelong avoidance of professional dental care, which can lead to significant untreated oral problems in adulthood (2).

To cope with stressful dental environments, a dynamic partnership among the child, the dental team, and the parents—called the pediatric dentistry treatment triangle—is considered necessary. According to the pediatric dentistry treatment triangle, a healthy relationship between the dental team and the parents can enhance the young patient’s attitude toward dental treatment (3). Parents serve as the primary gatekeepers, connecting the dental teams to young patients. Parents have an important role as both permission givers and co-decision-makers for young patients. While dentists are equipped with a continuum of behavior management techniques—ranging from basic communication to advanced pharmacologic interventions—the effectiveness of these methods is based on parental acceptance. Without parental trust and informed consent, even the most well-performed behavior strategies can fail.

BMTs may vary in their effectiveness depending on parental perceptions and contextual factors. The present study is grounded in three complementary theoretical frameworks. First, the Pediatric Dentistry Treatment Triangle conceptualizes the triadic relationship among the dentist, child, and parent as the foundational context for BMT effectiveness. Second, Baumrind's Parenting Typology (authoritative, authoritarian, and permissive parenting styles) explains differences in parental involvement and acceptance of communicative versus directive management strategies. Third, elements of the Health Belief Model explain how parental perceptions of dental treatment risks, perceived benefits, and self-efficacy influence their attitudes toward specific BMTs. Together, these frameworks support the hypothesis that parental sociodemographic and psychosocial characteristics are differentially related to acceptance of participatory (communicative) approaches compared with pharmacological or physical behavior management strategies (3).

In spite of the clinical importance of parental cooperation, the factors associated with their acceptance of various BMTs remain underexplored, particularly within the unique sociocultural landscape of Nakhon Ratchasima, Thailand. As the gateway to the northeast of Thailand, Nakhon Ratchasima encompasses diverse demographics, ranging from urban professionals to traditional rural caregivers. In Thailand, parenting paradigms have shifted (4) from conventional parenting styles highlighting responsiveness to more modern positive parenting styles. Contemporary Thai parents may have been evolving expectations regarding to how dental teams manage their children in the clinical environment (5).

Previous literature suggests that demographic and psychosocial backgrounds play an important role in shaping acceptance of each behavior management technique (6). For example, studies have demonstrated gender-based differences in anxiety levels (7, 8). Generational differences also play an important role in shaping each person's perspective. Digital natives across different generational cohorts often demand more transparency in professional interactions (9). However, whether these global trends apply to Thai parents living in Nakhon Ratchasima province remains unknown. Therefore, the purpose of our study was to determine the factors associated with parental acceptance of both basic and advanced behavior management techniques among Thai parents in Nakhon Ratchasima. Variables examined included age range, gender, educational level, dental anxiety, and parenting style. This research aims to provide dentists who work with young patients with the insights needed to customize their communication and clinical approaches, thereby fostering a harmonious and effective dental experience for young patients.

## 2 Materials and methods

This research employed a cross-sectional observational study design. Our study population comprised parents and primary caregivers of children residing in Muang district, Nakhon Ratchasima province, Thailand. Participants were recruited from parents or primary caregivers of children receiving dental treatment at the Oral Health Center of Suranaree University of Technology Hospital, and parents of preschool children attending accredited daycare centers and kindergartens in Muang District. Data collection was conducted between June and October 2025. Ethical approval for this research was granted by the Institutional Review Board of Suranaree University of Technology (SUT-IRB) [Certificate of Approval number only (COE No.) 111/2568]. The sample size was determined *a priori* using G*Power 3.1 software for a one-way analysis of variance (ANOVA), with the following parameters: effect size *f* = 0.25 (medium, per Cohen 1988), *α* = 0.05, statistical power = 0.80, and five comparison groups (age categories). This yielded a minimum required sample of *n* = 211 participants. The achieved sample of *n* = 215 slightly exceeded this threshold. It should be noted that the ≥60 years subgroup (*n* = 11, 5.1%) was substantially smaller than the other age cohorts; all comparisons involving this group are exploratory in nature and should not be generalized without replication in larger samples.

The participants were recruited through convenience sampling. Parents accompanying their children to pediatric dental offices at the study sites were consecutively approached according to the sampling procedure, informed about the study, and invited to participate in the study by trained members of the research team. Only those parents who fulfilled the inclusion criteria provided informed consent and were enrolled into the project.

Eligibility criteria included parents or primary caregivers of healthy children living in Muang district, Nakhon Ratchasima province, Thailand, who were identified as the child's primary caregiver, who had accompanied their child to a dental appointment, and who had previous experience with dental services. Exclusion criteria were parents or caregivers who had no previous dental experience, had never accompanied their child to a dental appointment, and caregivers of children with special healthcare needs.

### 2.1 Research questionnaire and data collection

Research questionnaires were distributed in both paper format and Google forms. Our study used a structured questionnaire divided into four primary sections.

The first section contained sociodemographic information of the parents/guardians, such as gender, age, relation to child, and education. The second section included a measure of parental dental anxiety through the Modified Dental Anxiety Scale (MDAS), originally developed and validated by Humphris et al. (10), with a Thai-language version validated for use in Thai dental patients by Klaophimai et al. (11). The third section comprised a Parenting Style Assessment based on the typological framework established by Baumrind (12) and operationalized using the Parenting Style Questionnaire developed by Robinson et al. (13), which was translated into Thai and validated to ensure linguistic accuracy and content validity. Prior to completing Section 4, each participant was provided with a standardized written description of each of the nine BMTs, accompanied by illustrative diagrams developed for lay audiences. The selection and classification of behavior management techniques followed the American Academy of Pediatric Dentistry (AAPD) Behavior Guidance for the Pediatric Dental Patient guideline (14).

Descriptions were reviewed for clarity and comprehensibility by a content validity committee and pilot-tested with 30 participants before data collection commenced. Verbal explanations by trained research assistants were also provided upon request. Participants were presented with two clinical scenarios:

Scenario 1: a non-invasive dental procedures or dental procedures performed without local anesthesia injection. Scenario 2: an invasive dental procedures or dental procedures performed with local anesthesia injection.

The study participants were informed of BMTs including Tell–Show–Do, Ask–Tell–Ask, Voice Control, Active Restraint, Passive Restraint, Parental Presence/Absence, Oral Sedation, and General Anesthesia. Participants rated their level of acceptance for each technique using a five-point Likert scale. Nitrous oxide sedation was excluded from the inferential analysis because it is not routinely in the clinical settings where the study participants receive care.

To calculate the overall acceptance score for each behavior management technique, the mean rating across the two clinical scenarios was computed to represent general parental acceptance. This manner of calculating acceptance has been used to measure the general acceptance or rejection rates without emphasizing scenario-specific variations.

### 2.2 Validation and reliability

Content validity was assessed by a committee of two pedodontists from the Dentistry Institute, Suranaree University of Technology. The Index of Item-Objective Congruence (IOC) was used to evaluate the relevance and clarity of each item (15). IOC scores for each item ranged from 0.67 to 1.00 (mean IOC = 0.84), with all items exceeding the minimum acceptable threshold of 0.60 (16). Recommendations and linguistic adjustments suggested by the committee were incorporated prior to full-scale data collection.

A pilot study was conducted with 30 participants using the same inclusion and exclusion criteria as the target sample. Internal consistency was confirmed using Cronbach's alpha coefficient (17), with a minimum acceptable threshold of 0.70. The following Cronbach's *α* values were obtained: MDAS Thai version *α* = 0.89 (15), Parenting Style Questionnaire *α* = 0.82, and BMT acceptance scale (overall) *α* = 0.78. All values exceeded the minimum threshold, confirming adequate internal consistency.

### 2.3 Statistical analysis

Data were analyzed using IBM SPSS Statistics version 26 and Microsoft Excel. The sociodemographic details of participants were summarized using descriptive statistics. Continuous variables were reported as mean ± standard deviation (SD) and categorical variables were expressed as frequencies and percentages.

Inferential statistical methods were employed to explore differences in parental acceptance scores of behavior management techniques based on sociodemographic characteristics. An independent-samples *t*-test was conducted to compare mean parental acceptance scores between male and female parents (Table 1). Utilizing a one-way ANOVA, we compared mean parental acceptance scores across four variables (parental age, level of education, parental anxiety regarding dental treatment, and parenting style), as shown in Tables 2–5. When a significant result was found in the overall ANOVA results, Tukey's HSD *post hoc* test was performed to compare the groups, with the results of those comparisons provided in Table 6.

**Table 1:** Comparison of parental acceptance by gender.

Technique	Male (mean ± SD)	Female (mean ± SD)	*p*-Value
Ask–Tell–Ask (with LA)	3.82 ± 0.71	4.12 ± 0.66	0.04
Tell–Show–Do	3.88 ± 0.74	4.03 ± 0.69	0.18
Parental Presence/Absence	3.59 ± 0.78	4.02 ± 0.72	0.03
Passive Restraint	3.55 ± 0.81	3.87 ± 0.75	0.09
Active Restraint	3.29 ± 0.86	3.37 ± 0.80	0.62
Voice Control	2.91 ± 0.83	3.13 ± 0.77	0.21
Oral Sedation	3.06 ± 0.79	2.82 ± 0.74	0.17
General Anesthesia	2.92 ± 0.91	2.57 ± 0.85	0.04

**Table 2:** Behavior management techniques according to parental age.

Technique	19–29 (mean ± SD)	30–44 (mean ± SD)	45–59 (mean ± SD)	>60 (mean ± SD)	*p*-Value
Ask–Tell–Ask	3.88 ± 0.69	3.86 ± 0.72	4.10 ± 0.67	3.71 ± 0.74	0.24
Tell–Show–Do	3.98 ± 0.73	3.73 ± 0.76	3.38 ± 0.82	3.00 ± 0.88	0.03
Passive Restraint	3.88 ± 0.77	3.43 ± 0.80	3.92 ± 0.74	3.37 ± 0.81	0.11
General Anesthesia	2.25 ± 0.84	2.51 ± 0.79	2.83 ± 0.73	2.86 ± 0.70	0.06

**Table 3:** Parental acceptance by education level.

Technique	Below bachelor’s (mean ± SD)	Bachelor’s (mean ± SD)	Above bachelor’s (mean ± SD)	*p*-Value
Ask–Tell–Ask	3.90 ± 0.70	4.21 ± 0.66	4.37 ± 0.61	0.04
Tell–Show–Do	3.75 ± 0.72	4.23 ± 0.68	4.42 ± 0.64	0.03
Parental Presence/Absence	3.82 ± 0.74	4.15 ± 0.69	3.84 ± 0.72	0.11
General Anesthesia	3.11 ± 0.83	2.95 ± 0.79	2.72 ± 0.76	0.09

**Table 4:** Sensitivity analysis of parental acceptance according to anxiety level.

Technique	No anxiety (*n* = 21) mean ± SD	Low anxiety (*n* = 82) mean ± SD	Moderate anxiety (*n* = 72) mean ± SD	High anxiety (*n* = 31) mean ± SD	*F*	*p*-Value
Ask–Tell–Ask	4.12 ± 0.65	4.08 ± 0.67	4.01 ± 0.69	3.83 ± 0.72	3.31	0.02
Tell–Show–Do	4.23 ± 0.63	3.94 ± 0.71	3.95 ± 0.70	3.92 ± 0.74	1.84	0.14
Parental Presence/Absence	4.01 ± 0.68	3.88 ± 0.72	3.93 ± 0.70	3.42 ± 0.77	3.78	0.01
General Anesthesia	3.07 ± 0.84	2.96 ± 0.79	3.08 ± 0.76	3.67 ± 0.73	2.14	0.09

Sensitivity analysis performed after excluding the very high anxiety subgroup (*n* = 9), which demonstrated zero within-group variance (SD = 0.00).

**Table 5:** Welch's ANOVA results.

Technique	Welch statistic	df1	df2	*p*-Value
Ask–Tell–Ask	4.12	3	47.8	0.01
Tell–Show–Do	1.62	3	46.9	0.19
Parental Presence/Absence	3.86	3	44.5	0.01
General Anesthesia	2.08	3	45.2	0.10

**Table 6:** *Post hoc* comparisons following sensitivity analysis.

Variable	Technique	Group comparison	Mean difference	95% CI	Adjusted *p*-value
Anxiety level	Ask–Tell–Ask	No vs. high	0.29	0.06–0.52	0.01
		Low vs. high	0.25	0.03–0.47	0.02
	Parental Presence/Absence	No vs. high	0.59	0.18–1.00	<0.001
		Low vs. high	0.46	0.10–0.82	0.02
		Moderate vs. high	0.51	0.13–0.89	0.01

Prior to conducting ANOVA, we verified that the required assumptions of normality and homogeneity of variance had been met by performing a Shapiro–Wilk test for normality and Levene's test for homogeneity of variance. To assess the robustness of our findings regarding parental dental anxiety, a sensitivity analysis was performed by re-running the one-way ANOVA without the very high anxiety group (*n* = 9), which exhibited zero within-group variance (SD = 0.00) for selected techniques.

In addition, Welch's ANOVA was performed as a secondary analysis due to its robustness to the assumption of equality of variances. Moreover, pairwise comparisons based on results from Welch's ANOVA were performed using the Games–Howell *post hoc* test when applicable. The findings from the primary ANOVA, sensitivity analysis, and Welch's ANOVA were compared to confirm that these relationships were stable and robust. All statistical tests were two-tailed, with significance set at *p*-value <0.05.

## 3 Results

Overall, 215 parents participated in this study (Table 7). Approximately 84% of respondents were women, whereas 15.8% were men. Half of the participants (50.0%) were in the 30–44 age group, followed by the 19–29 age group (19.5%), and the 45–59 age group (25.1%). The majority, or 47%, of respondents attained at least a bachelor's degree. Mothers made up the majority of the sample (73.5%). The most frequently reported levels of dental anxiety experienced by parents were low (38.1%) and moderate (33.5%); most parents (56.3%) chose the authoritative parenting style.

**Table 7:** Sociodemographic characteristics of the study participants (*n* = 215).

Variable	Category	Frequency (*n*)	Percentage (%)
Gender	Male	34	15.8
	Female	181	84.2
Age group (years)	19–29	42	19.5
	30–44	108	50.2
	45–59	54	25.1
	≥60	11	5.1
Education level	Below bachelor’s	79	36.7
	Bachelor’s	101	47.0
	Above bachelor’s	35	16.3
Relationship to child	Mother	158	73.5
	Father	43	20.0
	Guardian	14	6.5
Parental dental anxiety level	No anxiety	21	9.8
	Low anxiety	82	38.1
	Moderate anxiety	72	33.5
	High anxiety	31	14.4
	Very high anxiety	9	4.2
Parenting style	Authoritarian	64	29.8
	Authoritative	121	56.3
	Permissive	30	13.9

**Figure 1:**
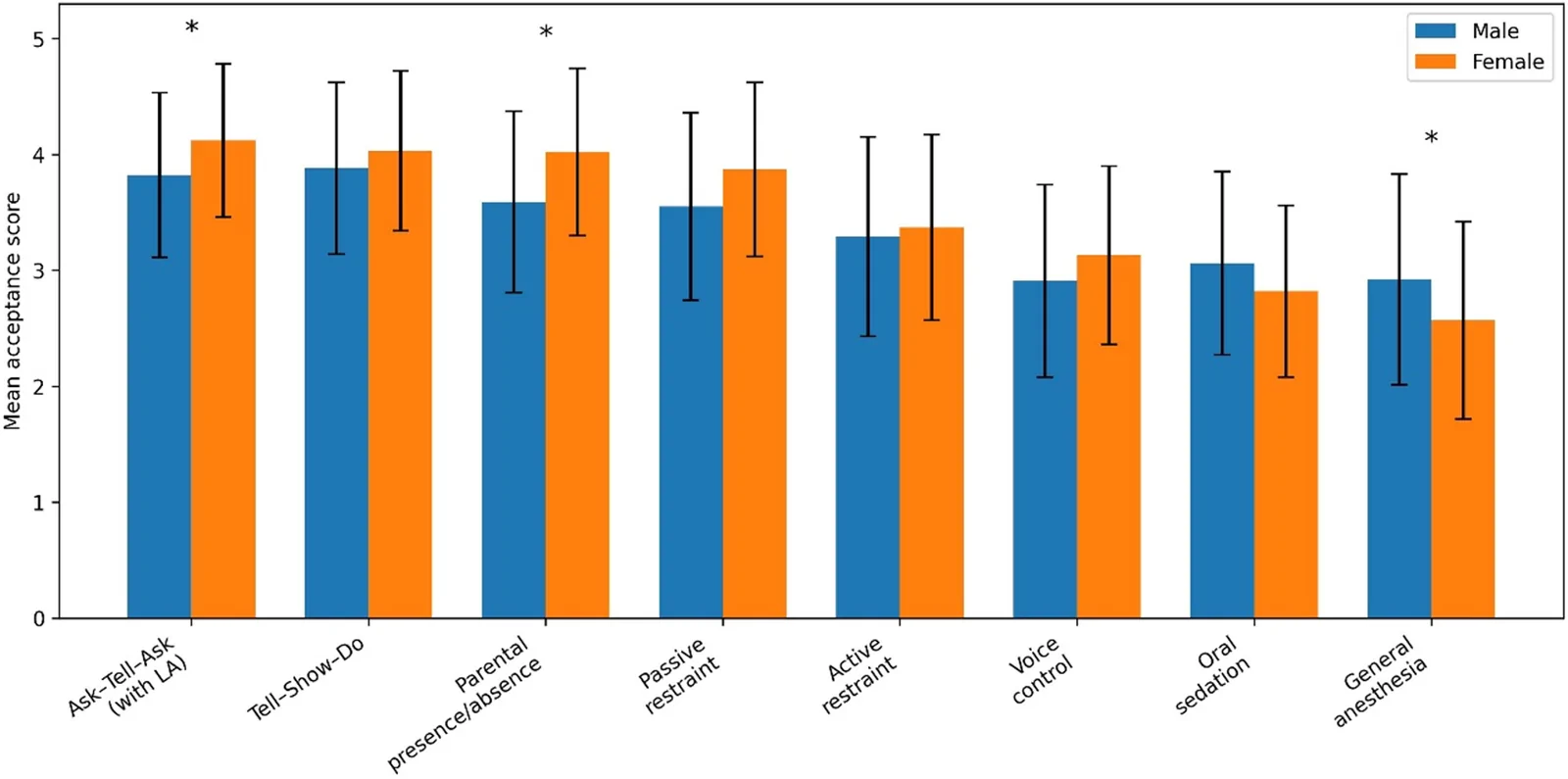
Mean acceptance scores for eight behavior management techniques (BMTs) stratified by parental gender.

Across all participants, the techniques with the highest mean acceptance scores were Tell–Show–Do (TSD) and Ask–Tell–Ask (ATA), reflecting a general parental preference for communicative and participatory approaches over pharmacological or physical restraint methods. Table 1 and Figure 1 present the gender-based analysis, examining parent acceptance of techniques and behaviors used with children experiencing dental pain and behaving poorly in the dental office environment. Women tended to give higher ratings on a greater number of the behavioral management strategies used than men. The Ask-Tell-Ask method (*p* = 0.04) and Parental Presence/Absence at the dental visit (*p* = 0.03) received significantly higher acceptance ratings among women than men. In contrast, acceptance of General Anesthesia varied significantly based on gender, with men reporting slightly higher acceptability ratings (*p* = 0.04). There was no evidence of statistically different gender effects on the acceptability ratings for Tell–Show–Do, Passive Restraint, Active Restraint, Voice Control, or Oral Sedation (*p* > 0.05).

The current research findings demonstrated a strong correlation between parental age and acceptance of the TSD technique as an intervention. The younger parent group (ages 19–29 years) showed much greater acceptance of TSD than other age groups, whereas the older parent group (age ≥ 60 years) showed the least acceptance. It should be noted that the ≥60 years group (*n* = 11) was substantially smaller than the other age cohorts, and all comparisons involving this group are exploratory in nature; results should not be generalized without replication in a larger purposive sample. Older parents tend to have lower acceptance of Tell-Show-Do compared to younger age groups but no statistically significant differences in acceptance for Ask-Tell-Ask, Passive Restraint, or General Anesthesia (Table 2).

Higher levels of parental education were significantly correlated with greater acceptance of the communicative behavior management techniques. Parents who received a higher level of education (greater than or equal to) than bachelor's degree had greater acceptance scores [for the Tell–Show–Do technique (*p* = 0.03) and the Ask–Tell–Ask technique (*p* = 0.04)]. There were no significant differences (*p* > 0.05) for acceptance of Parental Presence/Absence or use of General Anesthesia across education levels or groups (Table 3).

To assess the potential impact of the ceiling effect observed among those who reported very high levels of anxiety (*n* = 9), the data for the nine participants from this group were excluded in a sensitivity analysis. As shown in Table 4, after excluding those with a very high level of anxiety, significant differences in parental acceptance ratings remained present for Ask–Tell–Ask (*F* = 3.31; *p* = 0.022) and Parental Presence/Absence (*F* = 3.78; *p* = 0.012). In comparison, there were no significant differences found in ratings of Tell–Show–Do (*F* = 1.84; *p* = 0.141) and General Anesthesia (*F* = 2.14; *p* = 0.097).

In addition, Welch's ANOVA was conducted to evaluate the extent to which the findings from the sensitivity analysis could be corroborated due to the presence of unequal variances resulting from the subgroup with zero variance. The findings of Welch's ANOVA (*F*) were consistent with the results of the sensitivity analysis (Table 5). For Ask–Tell–Ask and Parental Presence/Absence, significant between-group differences were found (Welch statistic = 4.12, *p* = 0.011; Welch statistic = 3.86, *p* = 0.016, respectively); however, for Tell–Show–Do and General Anesthesia, no significant group differences were indicated (*p* = 0.196; *p* = 0.109, respectively).

After a detailed examination of the data for each of the *post hoc* outcomes, the results indicated that for children who had high anxiety, both the “Ask–Tell–Ask” and “Presence/Absence” models were significantly less accepted compared with children who did not experience any anxiety (all *p*-values were ≤0.05). Children who were classified as having low anxiety also had significantly lower levels of acceptance toward both of these models than their peers with no anxiety, although the average acceptance for these children was much closer to 0 when compared with children classified as having high anxiety. The difference in acceptance toward the “Ask–Tell–Ask” model between children with low anxiety and those with no anxiety was 0.25, which was not statistically significant.

Compared to parents with no dental anxiety, parents with high dental anxiety demonstrated significantly lower acceptance scores for participatory BMTs such as Ask-Tell-Ask and Parental Presence/Absence (Table 6).

The findings from the sensitivity analysis were consistent with the overall findings obtained from Welch's ANOVA, indicating that there were strong associations between parental dental anxiety levels and acceptance of various behavior management techniques, with minimal effect of excluding very high anxiety parents.

## 4 Discussion

The present study aimed to identify factors associated with parental acceptance of basic and advanced BMTs among Thai parents in Nakhon Ratchasima province. The findings demonstrated that parental gender, educational level, dental anxiety, and parenting style were significantly associated with the acceptance of communicative behavior management techniques. In contrast, parental age showed limited influence, with the exception of the TSD technique. Acceptance of pharmacological and physical restraint approaches appeared to be less influenced by the sociodemographic and psychosocial factors examined in this study. One of the most important outcomes in pediatric dentistry is helping patients cope with their fears. While many young patients are comfortable receiving dental treatment, some of them are frightened. If young patients experience anxiety or fear while receiving dental care, this can compromise the quality of the procedure and affect long-term prognosis. In addition, this kind of stress and anxiety may create a negative impression about dental care for the patient. These attitudes may foster lifelong avoidance in patients (18, 19), who may avoid routine dental check-up, leaving dental problems untreated. A good attitude toward dental treatment is the key to maintaining lifelong dental health in many patients. The pediatric dentistry treatment triangle comprises children, dental teams, and parents (3). Dentists are trained to employ many kinds of behavior management techniques, but the parental attitudes toward each behavior management technique are unknown. The purpose of our study was to investigate factors associated with parental acceptance of both basic and advanced behavior management techniques. The parental factors examined in this study included age range, gender, educational level, dental anxiety, and parenting style.

In general, women are1.5–2 times more likely to experience anxiety disorders than men (20). In our study, both genders preferred non-pharmacologic behavior management. Female parents demonstrated greater acceptance of participatory behavior management techniques such as Ask–Tell–Ask and Parental Presence/Absence compared with male parents. On the other hand, male parents reported higher acceptance of less participatory techniques such as General Anesthesia. It appears that mothers view their relationship with their child's dentist as one of collaboration; this means that mothers are looking to openly communicate and receive emotional support from their child's dentist. In contrast, further research is warranted to investigate whether paternal figures perceive the dental relationship differently, as our study design did not explicitly measure goal orientation constructs across genders.

Younger-generation parents value transparency and detailed explanations as a means to establish trust compared to older-generation parents (21, 22). On the contrary, older-generation parents value traditional institutional trust. Our findings indicated that there is a statistically valid association between the age range of a parent and their attitude toward the Tell–Show–Do technique. Parents in the 19–29 age range had higher acceptance scores than older groups, with the lowest level of acceptance for this technique occurring among those parents who were over 60 years of age. The higher acceptance of Tell–Show–Do among younger parents (19–29 years) might be explained by their exposure to modern pediatric healthcare models that emphasize transparency and psychological preparation. Digital-native parents might view the Tell–Show–Do technique as an instructional method that gives a sense of autonomy to their child and is more in line with current methods of positive parenting than traditional methods. The lower acceptance among older parents (>60 years) could signify generational differences regarding dental experiences, where older individuals may have encountered more traditional treatment styles and perhaps view the time and degree of detail required in the Tell–Show–Do technique as excessive (11, 23). However, a major concern arises when interpreting the association between parental age and BMT acceptance. The group of parents aged over 60 years in our study was small (5.1% of our sample). Therefore, the result showing lower acceptance of the Tell–Show–Do technique must be interpreted with extreme caution, and further investigation is required to validate these generational trends. Most behavior management techniques showed limited association with parental age. These results imply that while basic communication (Ask–Tell–Ask) is valued by parents of all ages, the lack of significant age-based differences in the acceptance of advanced techniques such as General Anesthesia suggests that certain methods possess a universal clinical appeal. For example, General Anesthesia may be accepted across all age groups as a definitive solution for complex cases, regardless of the parent's generational background. Similarly, the neutral response toward Passive Restraint across all ages indicates that when a technique is perceived as a safety necessity or a highly specialized intervention, parental age becomes a less decisive factor than clinical urgency.

Our findings show that parents with at least a bachelor's degree have higher acceptance scores for Tell–Show–Do and Ask–Tell–Ask. Both require interaction between dentists and children. This correlation strongly highlights the role of health literacy and information-seeking behavior among well-educated people Prior research had demonstrated strong associations between higher educational levels, health literacy, and health information-seeking behaviors (24, 25). Parents with higher educational attainment are more likely to be familiar with environments that prioritize detailed explanations and participation. Consequently, they may expect dentists to apply the same educational, step-by-step approach with their children, viewing reciprocal communication as an essential component of high-quality dental care (26). On the other hand, there is no significant association between parent's educational level and their acceptance of General Anesthesia or Parental Presence/Absence. If a child's dental condition is severe or complex and requires General Anesthesia, parents appear to choose based on safety necessity rather than elective preference. Similarly, the decision to remain in the dental room may be driven by different variables other than the parent's educational level (13).

Parental dental anxiety is associated with children's level of cooperation during treatment (27). Children often reflect their parents’ fears, which may lead to uncooperative behaviors, such as avoidance, tantrums, and crying. Our findings suggest that those parents with lower dental anxiety often seek a higher degree of involvement; our results show that parents with low or no dental anxiety have higher acceptance scores for participatory behavior management techniques such as Ask–Tell–Ask and Parental Presence/Absence. Parents with higher dental anxiety seem to separate themselves from dental involvement and prefer the dentist to make the decisions. Due to the importance of the caregiver role, which may be associated with children's fears, dentists need to manage and address parental anxiety prior to dental treatment and clarify the parent’s role as an observer (15).

Parenting styles are determined by levels of responsiveness and demandingness (28). The authoritative parenting style is characterized by high responsiveness and high demandingness, the authoritarian parenting style by low responsiveness and high demandingness, and the permissiveness parenting style by high responsiveness and low demandingness. Our findings revealed that authoritative parents had the highest acceptance score for every behavior management technique. The significant relationship between parenting styles and the acceptance of management techniques emphasizes the importance of parental expectations in dental settings. The philosophy of authoritative parenting style aligns with the Ask–Tell–Ask technique and is an extension of the home environment where authoritative parents allow their children to ask questions. Similarly, Parental Presence suggests the desire of parents to serve as a secure base for their children, while Parental Absence allows their children to develop independent coping skill (29). Although both authoritarian and permissive parents showed lower acceptance of participatory behavior management techniques, the reasons were different. Authoritarian parents may perceive that these BMTs are less efficient or misaligned with their directive parenting style, while permissive parents try to avoid establishing boundaries for their child and tend to fail after their children express fears.

Our findings show that parents in Nakhon Ratchasima demonstrate greater acceptance of communicative and participatory behavior management techniques compared with pharmacological or other advanced behavior management techniques. These findings are broadly consistent with previous studies conducted in Saudi Arabia, Brazil, India, and the United States of America (6, 30, 31). These similarities across diverse cultural settings suggest that communication, transparency, and child participation during dental treatment are highly valued by parents worldwide. Nevertheless, cultural differences may still shape parental preferences. Previous studies have emphasized the role of sociocultural norms, parental involvement in healthcare decision-making, and expectations regarding child autonomy in influencing the acceptance of specific behavior management techniques (32). Various factors were associated with the acceptance of each behavior management technique. Dentists should customize the behavior management techniques used for each young patient. Our findings confirm that no single technique is universally superior. Pediatric dentists should select appropriate techniques tailored to individual family profiles to foster trust and coping skills.

### 4.1 Clinical implications and future directions

- Improve the way dentists communicate with parents. Dentists should clarify to parents the methods used to manage children's behaviors, particularly the communications approach through Tell–Show–Do and Ask–Tell–Ask techniques.
- Parental characteristics are closely associated with levels of acceptance of behavior management techniques. By understanding parental factors such as education level and anxiety toward dentistry, the dentist can develop a treatment plan and determine the appropriate approach to use to manage the child’s behavior.
- Parental anxiety related to their child's treatment must be assessed before the provision of any services. A parent's perception of their child's treatment can have an effect on their child's behavior throughout the appointment.
- When parents wish to be more actively involved in their child's treatment, they are likely to cooperate with and support the dentist in implementing portions of the treatment plan.
- Future studies should examine larger and more diverse samples across multiple regions of Thailand to provide a better understanding of the relationship between cultural factors and how parents view behavior management techniques in the field of dentistry.

The following guidelines outline the best practices for engaging with parents in order to improve children's cooperation during dental appointments.

### 4.2 Limitations

The following limitations should be considered when interpreting the findings of this study:

Cross-sectional design: The study employed a cross-sectional design, which precludes causal inference. The associations reported between parental characteristics and BMT acceptance reflect correlational relationships only and should not be interpreted as evidence of causation.
Gender imbalance in the sample: Female respondents comprised 84.2% of the sample, reflecting the real-world composition of primary dental caregivers at the data collection sites. Gender-specific findings should be interpreted with this sampling bias in mind, and conclusions regarding male parents should not be overgeneralized.
Geographic restriction: Data were collected exclusively within Nakhon Ratchasima province. As a result, findings may not be generalizable to other Thai provinces, urban metropolitan centers, or non-Thai populations.
Self-report bias: Dental anxiety and parenting style were assessed via self-administered questionnaires, which are subject to social desirability effects. Observed parental behavior during actual dental appointments was not assessed.
Small ≥60 years’ subgroup: The oldest age group contained only 11 participants (5.1%), rendering all comparisons involving this group statistically underpowered. These results are exploratory and require replication in larger purposive samples.

One important limitation of this research is that the analyses were carried out using only bivariate comparisons, without the use of multivariable regression to account for confounding variables. This means that the results shown cannot be seen as independent and must be considered in the broader context in which the factors of parental age, education, dental anxiety, parenting style, and child–parent relationships may interact. The results presented should be regarded as exploratory, offering opportunities for hypothesis creation rather than being interpreted as evidence of independent relationships between variables. Future research should employ larger samples of participants and multivariable regression models in order to find independent variables affecting parental acceptance of behavior management techniques.

## 5 Conclusion

This study explored various factors associated with Thai parents’ acceptance of BMTs in pediatric dentistry in Nakhon Ratchasima, Thailand. Parental acceptance of BMTs was associated with several demographic (or non-demographic) and psychosocial characteristics such as parent's gender, education level, dental anxiety, and parenting style. Female parents were more accepting of participatory communication-based BMTs (e.g., Ask–Tell–Ask and Parental Presence/Absence) than male parents, who had a higher level of acceptance of General Anesthesia. In addition, parents with higher levels of education had a higher level of acceptance for communicative BMTs (e.g., Tell–Show–Do and Ask–Tell–Ask) than those with lower levels of education. Parents who had high levels of dental anxiety and parents with an authoritative parenting style demonstrated greater acceptance of interactive/participatory BMTs. Parental age only showed a small effect overall, although younger parents had a greater level of acceptance of the Tell–Show–Do technique.

Parental perceptions of behavioral management strategies are associated with many different things, including their sociodemographic and psychological makeup. By understanding the factors that shape parental expectations, pediatric dentists can design more effective behavioral guidance strategies and acquire clearer communication with caregivers. If dentists design their behavior management strategies to align with parental expectations, it will generally lead to improved cooperation among very young children, lower stress/anxiety levels on the part of the children, and positive dental experiences.

## Data Availability

The raw data supporting the conclusions of this article will be made available by the authors, without undue reservation.
